# The usefulness of super-selective arterial spin labeling for postoperative evaluation of pediatric moyamoya disease: technical note

**DOI:** 10.1007/s00234-024-03402-2

**Published:** 2024-06-13

**Authors:** Tsutomu Yoshikane, Kentaro Hayashi, Makoto Obara, Takeshi Katsube, Hiroya Asou

**Affiliations:** 1 Department of Neurosurgery, Shimane University Faculty of Medicine, 89-1 Enya, Izumo, Shimane 693-8501 Japan; 2 MR Clinical Science, Philips Japan Ltd., Tokyo, Japan; 3 Department of Radiology, Shimane University Faculty of Medicine, Izumo, Japan

**Keywords:** MR technique, Arterial spin labeling, Super-selective ASL, Moyamoya disease, Pediatric radiology

## Abstract

Moyamoya disease is characterized by progressive internal carotid artery (ICA) occlusion. Extracranial-intracranial bypass surgery is effective, particularly in pediatric patients; imaging plays a crucial role in evaluating intracranial perfusion pre- and post-surgery. Arterial spin labeling (ASL) is a magnetic resonance technique employed for noninvasive, whole-brain perfusion assessment by magnetically labeling inflowing blood. However, ASL cannot evaluate the territories and development of each vessel perfusion compared with digital subtraction angiography (DSA). Recently, super-selective ASL (SS-ASL) has been developed, performing pinpoint labeling on a specific artery at a time, and offering a tomographic view that distinctly displays blood supply areas for each vessel. Unlike DSA, SS-ASL is noninvasive and can be repeatedly performed in pediatric patients. In conclusion, SS-ASL is useful for evaluating bypass development over time and understanding the pathophysiology of pediatric moyamoya disease.

## Introduction

Moyamoya disease (MMD) is characterized by steno-occlusive lesions in the terminal portion of the bilateral internal carotid arteries (ICAs) and the development of compensatory fine and fragile arteries [1, 2]. This disease is predominantly found in East Asian countries, with an incidence of 0.54/100,000/year [3]. Diagnosis often relies on ischemia in children. Vascular reconstruction surgery such as extracranial to intracranial (EC-IC) bypass is recommended in symptomatic patients. Furthermore, 6.8% of pediatric patients with MMD exhibit progressive posterior cerebral artery (PCA) stenosis and require bypass surgery [4], necessitating, postoperative assessment of cerebral hemodynamics. Although digital subtraction angiography (DSA) and/or magnetic resonance angiography (MRA) are commonly employed for this purpose, invasiveness and radiation exposure make DSA less suitable for pediatric patients. Though MRA is noninvasive, it does not show the perfusion territory in details. Recently, sonography has been reported to be useful for assessing bypass function easily and noninvasively; however, the details are difficult to assess [5]. Arterial spin labeling (ASL) is a magnetic resonance (MR) imaging technique used for evaluating cerebral perfusion noninvasively by magnetically labeling the inflowing blood. However, standard ASL can only visualize perfusion in the whole brain. Recently, super-selective ASL (SS-ASL) has been developed and can label separately into the external carotid artery (ECA), ICA, and vertebral artery (VA). This technique uniquely enables rapid and non-invasive visualization of each vascular territory on tomographic images [6, 7]. Herein, we report the usefulness of SS-ASL imaging for evaluating cerebral perfusion post-vascular reconstruction surgery in pediatric patients with MMD.

## Methods

### MR imaging

**Fig. 1:**
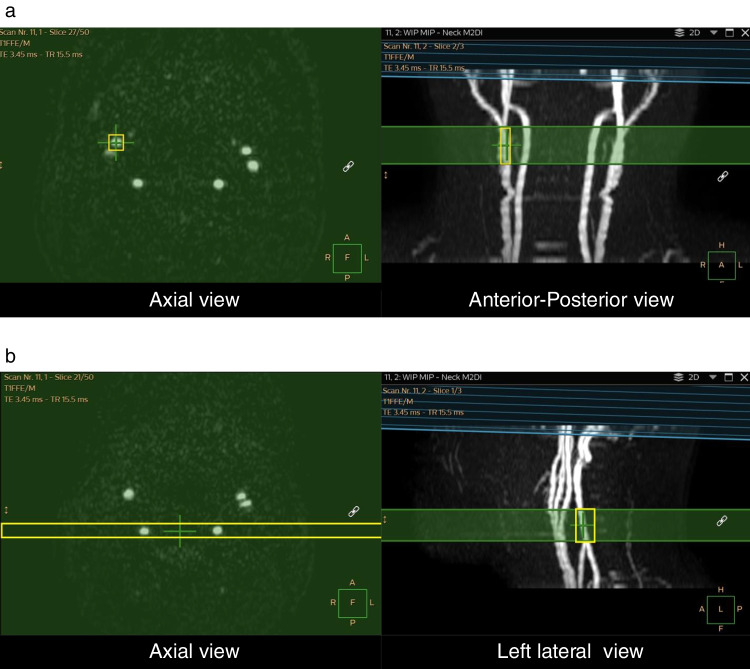
Planning steps of superselective arterial spin labeling (SS-ASL) sequence. The labeling stack is positioned on a Three-Dimensional Time-of-Flight Magnetic Resonance Angiography (3D TOF MRA) of the cervical vessels (e.g. Right external carotid artery (ECA) (**a**) and bilateral vertebral arteries (VAs) (**b**)). Labeling locations are shown in yellow boxes

MR imaging was performed using a 3 T whole-body system (Ingenia Elition; Philips Healthcare, Best, Netherlands) with a whole-body radiofrequency coil for transmission and a 32-channel phased array head coil for signal reception. Scanning and tagging parameters of the SS-ASL adhered to the technical guidelines in principle [8, 9]. These parameters at our facility were: FOV 240 × 240 mm, voxel size of 2.5 × 2.6 × 10 mm^3^, 3D fast field echo-planar imaging readout, TR/TE/FA = 4220 ms/18 ms/90-degree, labeling duration of 1.80 s, post labeling delay of 2.20 s, with background suppression consisting of 4 pulses after a saturation pulse that preceded the labeling. Fourteen slices of labeled and control images were acquired at a scan time of approximately 2 min. The scan plane was angulated in the same manner as the labeling plane. In the planning steps of SS-ASL, the labeling stack was initially positioned on a 3D time-of-flight (TOF) MRA image of the cervical vessels. The circular labeling spot was adjusted in the graphic user interface to cover the target vessel in its craniocaudal extension of at least 20 mm (Fig. 1a). Regarding the posterior circulatory perfusion specifically, labeling of the bilateral VAs was simultaneously performed (Fig. 1b). The diameter of the circular label focus was set to 8 mm. Second, the readout slab was positioned to cover the entire brain perfusion territory [10]. If patient movement was detected during examination, TOF scan was repeatedly performed, the labeling locations were updated directly before performing the SS-ASL sequence to minimize the loss of labeling efficiency due to motion.

### Case presentation

**Fig. 2:**
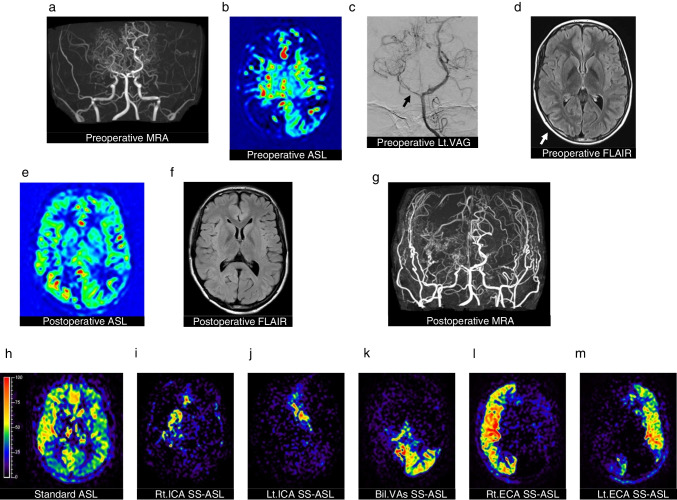
(**a**) Preoperative magnetic resonance angiography (MRA) demonstrating multiple changes in arterial vascular calibers and (**b**) Preoperative arterial spin labeling (ASL) showing reduced cerebral blood flow. (**c**) Preoperative left (Lt.) vertebral angiography (VAG) showing right posterior cerebral artery (PCA) stenosis (arrow) and (**d**) Preoperative Fluid-attenuated inversion recovery (FLAIR) showing ivy sign (arrow). (**e**) Postoperative ASL showing improved blood flow in the whole brain and (**f**) Postoperative FLAIR showing disappearance of ivy sign and no abnormal findings. (**g**) MRA showing excellent development of the bypasses from the bilateral external carotid artery (ECA). (**h**) Standard ASL showing improved blood flow in the whole brain (in ml/100 g/min). (**i**, **j**) superselective-ASL (SS-ASL) showing that each internal carotid artery (ICA) supplies the ipsilateral basal ganglia, (**k**) bilateral vertebral arteries (VAs) supply the left occipital lobe, (**l**) the right ECA supplies cortical areas of the anterior and posterior circulation in the right cerebral hemisphere and (**m**) the left ECA supplies cortical areas of the left anterior circulation including the right posterior circulation

A 9-year-old girl presented with stage IV MMD (Fig. 2a). Preoperative standard ASL showing reduced cerebral blood flow in bilateral cerebral hemispheres (Fig. 2b). First, bypass surgery was performed bilaterally for anterior circulation. Two years later, progression of the right PCA stenosis caused the appearance of ivy signs indicating ischemia in the right occipital lobe, and bypass surgery was performed for the right PCA territory (Fig. 2c, d). The patient’s postoperative course was uneventful (Fig. 2e, f). Three years post-initial surgery, MRA showed progression of bilateral ICA stenosis and right posterior cerebral artery occlusion, as well as excellent development of the bypasses from the bilateral ECA (Fig. 2g). Standard ASL showed improved blood flow in the whole brain (Fig. 2h). SS-ASL showed areas of blood supply from each vessel separately on the tomographic view (Fig. 2i-m).

## Discussion

**Fig. 3:**
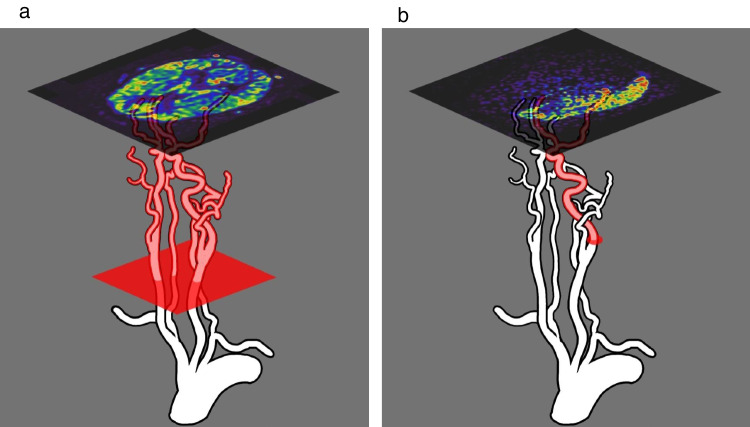
Schematic diagram showing the difference between standard arterial spin labeling (ASL) and superselective-ASL (SS-ASL). The red area in the diagram shows the labeling area. (**a**) standard ASL can only evaluate cerebral perfusion in the whole brain because it labels the entire axial section of the cervical vessels in the neck. (**b**) SS-ASL labels a specific artery and shows the cerebral circulation supplied by the selected artery

Postoperative cerebral perfusion is evaluated with single photon emission computed tomography (SPECT), positron emission tomography (PET), computed tomography (CT) perfusion, DSA, and ASL [1, 11]. Although SPECT, PET, CT perfusion, and DSA involve radiation exposure and SPECT, PET, and CT perfusion could only evaluate perfusion in the entire brain, DSA is an ideal modality for assessing the territory and development of bypass perfusion. However, it is not suitable for evaluating changes over time because it cannot be performed frequently due to the risks associated with the procedure, such as cerebral infarction [12]. Furthermore, pediatric patients sometimes require general anesthesia to maintain their posture during DSA. ASL is a safe and universal examination method that can evaluate cerebral perfusion without radiation exposure or contrast medium because it uses protons in arterial blood as endogenous tracers using radio waves to label them magnetically and is also suitable for follow-up. However, ASL can only evaluate cerebral perfusion in the whole brain because it labels the entire axial section of the cervical vessels in the neck and cannot evaluate cerebral perfusion in individual arteries, as DSA can. By contrast, SS-ASL labels a specific artery and shows the cerebral circulation supplied by the selected artery (Fig. 3). Hwang et al. reported that SS-ASL findings were consistent with those of DSA [13]. In the case presented here, SS-ASL clearly evaluated perfusion and showed that the cortical areas of the bilateral anterior and right posterior circulation received perfusion from their associated ECAs. Using DSA, taking images of each vessel to evaluate the area of blood supply is necessary, which can also be evaluated by comprehensively assessing all image findings obtained by taking images from several angles. This requires advanced anatomical knowledge and abundant experience. By contrast, SS-ASL can evaluate the perfusion profiles of each vessel in the whole brain on tomographic images. Therefore, visually evaluating the perfusion territory is easy not only in the cortex but also in the medulla, such as the basal ganglia and corona radiata. SS-ASL takes only approximately 2 min per vessel using our method and can be performed during an outpatient visit, making evaluating cerebral hemodynamic changes over time possible and being useful for understanding the pathophysiology of pediatric MMD.

There are some limitations in the present study. First, movement was a significant limitation in SS-ASL acquisition due to the need for precise placement of the labeling volumes. Although this might be problematic in general patient populations, it could be much more problematic in pediatric cases. However, although mild sedation was required to adequately perform MR imaging in most of pediatric patients, none of the cases required general anesthesia. Furthermore, SS-ASL takes only approximately 2 min per vessel using our method, the rate of failure in our institutions is as low as 1 in 125 cases (data not shown). Second, turbulent flows may have decreased labeling efficiency or caused signal inhomogeneities during readout. Additionally, the lumen size of the labeled structure could have influenced perfusion related signal changes in the readout volume. Lastly, although diagnostic worth was not excluded, ASL could be dependent on heart rate, blood pressure, hematocrit, blood CO2 levels, and blood flow velocity at the time of the imaging. Therefore, ASL could not quantitatively assess cerebral blood flow. As a result, a correction method should be developed and optimized to evaluate with high reproducibility.

## Conclusions

SS-ASL is a unique modality that could show the blood supply areas from each vessel separately on tomographic view. In addition, its noninvasive nature could allow for repeated use in children, making it useful as a postoperative evaluation method for cerebral hemodynamics in pediatric patients with MMD.

## Data Availability

The data that support the findings of this study are available from the corresponding author upon reasonable request.

## Abbreviations

ASL: Arterial spin labeling
Bil: Bilateral
CT: Computed tomography
DSA: Digital subtraction angiography
ECA: External carotid artery
FLAIR: Fluid-attenuated inversion recovery
ICA: Internal carotid artery
PCA: Posterior cerebral artery
PET: Positron emission tomography
SPECT: Single photon emission computed tomography
VA: Vertebral artery
VAG: Vertebral angiography
